# Ischemic stroke in different ICU types

**DOI:** 10.1186/cc12435

**Published:** 2013-03-19

**Authors:** M Damasceno, F Rodriguez, R Turon

**Affiliations:** 1 Hospital de Clinicas de Niterói, Rio de Janeiro, Brazil

## Introduction

In our hospital there are a general intensive care unit (GICU) and a neurointensive care unit (NICU). Despite the preference for the NICU, in both there are admissions for ischemic stroke. There are different staff for each ICU, with the same physician's leadership. We have decided to evaluate the performance of both ICUs, analysing whether there are differences in results as some authors publish best results in specialized ICUs.

## Methods

Using prospectively collected data, we undertook a retrospective evaluation of all patients admitted to the GICU and NICU of our hospital with the diagnosis of ischemic stroke, from December 2010 to November 2012. In both ICUs there are intensivists, but in the NICU the intensivists have special expertise in neuroscience. Data were collected from Epimedmonitor.

## Results

A total of 3,854 admissions were registered in the period in both ICUs, with 257 (6.7%) being by ischemic stroke - 49 in GICU and 208 in NICU. Mean age of patients: 73 years in GICU and 70.1 years in NICU. Admissions from emergency unit: 44 (89.8%) GICU, 181 (87%) NICU. Mean SAPS 3 score: 50.7 (29 to 71) GICU, 50.6 (29 to 98) NICU. Patients admitted with infection: 6 (12.2%) GICU, 10 (4.81%) NICU. Mean Charlson comorbidity index points: 1.61 (median 1.0) GICU, 1.1 (median 1.0) NICU. Mean length of ICU stay: 4.8 days (median 3) GICU, 4.3 days (median 3) NICU. Mean length of hospital stay: 12.2 days (median 8) GICU, 14.5 days (median 8) NICU. Predicted hospital mortality (mean ± SD): 20.07% ± 13.48 GICU, 20.4% ± 13.83 NICU. Hospital mortality rate: 12.5% GICU, 5.39% NICU. Observed-to-expected (O/E) mortality ratios: 0.62 GICU, 0.26 NICU.

## Conclusion

Despite the similar proportions numbers for patients in both ICUs, the mortality rate and the O/E mortality ratio for ischemic stroke were higher in patients of the GICU when compared with the NICU.
